# Sociodemographic and Health Correlates of Health-Promoting Lifestyle Behaviors Among Nursing Students

**DOI:** 10.3390/nursrep16050150

**Published:** 2026-04-23

**Authors:** Itziar Hoyos Cillero, Iñigo Lorenzo Ruiz

**Affiliations:** Department of Nursing I, Faculty of Medicine and Nursing, University of the Basque Country (UPV/EHU), Barrio Sarriena SN, 48940 Leioa, Spain; inigo.lorenzo@ehu.eus

**Keywords:** nursing students, health promotion, Mediterranean diet, physical activity, nursing curriculum

## Abstract

**Background/Objectives:** Limited research has examined the correlates among the lifestyle habits of nursing students, whose suboptimal behaviors may compromise their ability to model and promote healthy lifestyles in future professional practice. This study aimed to assess health-promoting lifestyle behaviors, explore interrelationships among lifestyle domains, and identify key correlates of positive health-promoting lifestyle behaviors to inform the development of targeted interventions. **Methods:** A cross-sectional study was conducted among 476 undergraduate nursing students in Spain. Data included sociodemographic, academic, and health-related variables, along with Health-Promoting Lifestyle Profile II (HPLP-II) scores. Descriptive statistics, correlations, and hierarchical multivariate logistic regression were used to identify factors associated with positive health-promoting lifestyle behaviors. **Results:** Overall HPLP-II scores indicated modest health-promoting lifestyle behaviors (adjusted mean 2.62 ± 0.33), with the lowest scores observed for health responsibility (adjusted mean 2.20 ± 0.48) and stress management (adjusted mean 2.33 ± 0.44). Health-related variables showed stronger associations with positive health-promoting lifestyle behaviors than sociodemographic or academic variables (*p* < 0.001). Significant correlates of positive health-promoting lifestyle behaviors included higher adherence to the Mediterranean diet, greater levels of physical activity, and concurrent employment during studies. **Conclusions:** Support of nutrition, physical activity, and other health-promoting lifestyle behaviors should be strengthened in nursing curricula and training environments. Educational strategies should move beyond theoretical instruction through student-centered approaches, enhancing self-care and the ability to promote health in future professional practice.

## 1. Introduction

Nurses constitute the largest workforce in the health sector, accounting for approximately 59% of all health professionals worldwide [1]. The 2025 International Council of Nurses definition emphasizes nurses’ role as agents of change in promoting health and building sustainable, equitable health systems, with a particular focus on health promotion to improve overall well-being [2]. Non-communicable diseases (NCDs) are the leading cause of death globally [3] and in Spain [4], highlighting the need for effective lifestyle interventions [3,4,5]. University students, including nursing students, face academic and clinical demands that can foster unhealthy behaviors [6,7]. Although they receive education on health and disease prevention, many exhibit suboptimal health behaviors, such as insufficient physical activity, poor diet, inadequate stress management, and low health accountability [8,9,10,11], despite understanding their importance. Promoting a health-enhancing lifestyle during undergraduate education is essential to prevent future health problems and reduce professional burnout [12,13,14].

The Health Promotion Model (HPM) provides a widely used framework for understanding health-promoting lifestyle behaviors, emphasizing the proactive role of individuals and the interaction between personal, interpersonal, and environmental factors [15]. Within the model, a health-promoting lifestyle—including responsible health practices, physical activity, nutrition, stress management, spiritual growth, and positive relationships—is a key determinant of well-being [15,16]. Based on the HPM, the Health-Promoting Lifestyle Profile II (HPLP-II), developed by Walker et al. (1987), has been validated internationally, demonstrating reliability and relevance for both clinical and research purposes [17,18,19,20,21]. International studies have linked HPLP-II scores in nursing students to personal, academic, and health-related factors, highlighting areas for intervention to improve student well-being [9,10,11,12].

Despite the importance of health-promoting lifestyle behaviors, research on nursing students in Spain is limited, particularly regarding their engagement with the HPLP-II. While studies in other Mediterranean countries have examined these behaviors in nursing students [9,22,23,24,25,26,27], few have simultaneously considered a comprehensive set of variables—including sociodemographic, academic, and health-related factors—alongside adherence to the Mediterranean diet using the KIDMED questionnaire [28] and physical activity levels assessed by the International Physical Activity Questionnaire (IPAQ) [29]. To our knowledge, no study in Spain has systematically analyzed these relationships. This is essential to identify context-specific factors influencing health-promoting lifestyle behaviors and to inform tailored interventions within the Spanish context.

Addressing this gap, the present study aimed to accomplish the following:

(1) Examine HPLP-II scores across all six dimensions and their intercorrelations.

**Hypothesis** **1:** *The six HPLP-II subscales are positively intercorrelated, reflecting the multidimensional but interconnected nature of health-promoting lifestyle behaviors*.

(2) Analyze the relationships between sociodemographic–academic and health-related variables and health-promoting lifestyle behaviors through comparative analysis.
(3) Identify the strongest correlates of these behaviors using hierarchical regression models.

**Hypothesis** **2:** *Health-related factors are more strongly associated with higher HPLP-II scores and have greater predictive capacity than sociodemographic–academic factors*.

Based on the HPM, the model used in this study was operationalized as follows: personal factors included sociodemographic variables (sex and age), academic characteristics (year of study and employment status), and individual health-related factors (presence of chronic disease and awareness of health-promoting lifestyles); interpersonal and environmental influences were represented by sociodemographic variables reflecting the family and living context, including parental education, parental employment status, family structure, habitual residence, and place of residence; and health-related behaviors included physical activity, adherence to the Mediterranean diet, sleep duration, smoking status, screen time, BMI, prior experience in health-related fields, and engagement in university wellness activities. Finally, health-promoting lifestyle behaviors, the primary outcome, were assessed using the HPLP-II.

## 2. Materials and Methods

### 2.1. Study Design and Setting

A cross-sectional study was conducted from October to November 2023 among nursing students at the Faculty of Medicine and Nursing of the University of the Basque Country (Leioa, Spain).

### 2.2. Study Sample

A total of 660 first-to-fourth-year undergraduate nursing students were invited to participate in the study (163 first years, 160 s years, 167 third years, and 167 fourth years). Participants who provided informed consent and had no conditions that could affect their lifestyle (e.g., multiple sclerosis or musculoskeletal disorders) were included. The presence of such conditions was self-reported via a question included in the questionnaire.

A standard formula for estimating proportions to determine the mean HPLP-II score was used, with a 95% confidence level (Z = 1.96), a conservative prevalence estimate (*p* = 0.50) that maximized variance [30], and a ±5% margin of error (d = 0.05), yielding a sample size of 385 students. The final target was adjusted upward to 424 participants to account for an anticipated 10% non-response rate. Ultimately, 476 students participated, exceeding the planned sample size due to higher-than-expected consent during recruitment.

### 2.3. Study Instruments

Participants completed a questionnaire consisting of two sections. The first section gathered data on sociodemographic, academic, and health-related characteristics. Sociodemographic and academic variables included sex (female or male), age, year of study (1st, 2nd, 3rd, or 4th), maternal and paternal educational levels (primary, secondary, or higher), parental employment status (working: yes or no), family structure (monoparental or biparental), place of residence (urban or rural), type of habitual residence (family home or flat renting or residence), and employment during studies (yes or no).

Health-related characteristics included prior work experience in health-related fields before entering university (yes or no), engagement in university wellness activities (e.g., sports and health education sessions) (yes or no), awareness of health-promoting lifestyle behaviors (yes or no), the presence of any chronic disease not limiting lifestyle (yes or no), smoking status (yes or no), and daily sleep duration. Self-reported sleep time was recorded in hours and minutes and dichotomized as ≥7 h/day or <7 h/day, in accordance with expert recommendations for adults [31]. Screen-viewing activities were also assessed by asking students how much time they typically spend on various devices—smartphone, TV, game console, tablet, and computer—on a usual weekday and weekend day. Total screen time for weekdays and weekends was calculated by summing all reported usage and dividing by 7 (weekdays) or 2 (weekend days). Responses were then categorized according to the Canadian 24-Hour Movement Guidelines for adults (18–64 years), which recommend no more than 3 h/day of recreational screen time [32]. Participants were classified as either meeting the guideline (≤3 h/day) or exceeding it (>3 h/day). These internationally recognized guidelines provide a consistent framework for analyzing screen-time behaviors in our university population. Finally, mean daily sleep duration and average screen-viewing time were computed for analysis.

Participants self-reported their weight and height, from which Body Mass Index (BMI, kg/m^2^) was calculated. BMI categories (underweight: <18.5 kg/m^2^; normal: 18.5–24.9 kg/m^2^; overweight: 25–29.9 kg/m^2^; obese: ≥30 kg/m^2^) were assigned following the Centers for Disease Control and Prevention standards [33]. Adherence to the Mediterranean diet was assessed using the 16-item KIDMED questionnaire [28]. Results were categorized as high (≥8 points), medium (4–7 points), or low adherence (≤3 points), based on the total score obtained. Physical activity habits were evaluated using the short form of the IPAQ, a validated tool widely used in adult populations [29]. Obtained results were classified as low, moderate, or high physical activity levels, according to the official IPAQ scoring protocol [34].

The second section of the questionnaire included the HPLP-II, used to assess health-promoting lifestyle behaviors [17]. This 52-item instrument is organized into six subscales: health responsibility (nine items), physical activity (eight items), stress management (eight items), nutrition (nine items), spiritual growth (nine items), and interpersonal relationships (nine items). Participants respond using a 4-point Likert scale: never (1), sometimes (2), often (3), and routinely (4). The overall HPLP-II score is calculated by averaging the responses across all 52 items, resulting in a score ranging from 1 to 4 (equivalent to a total score of 52–208). Subscale scores are obtained similarly by averaging the responses to the items within each subscale. In line with previous studies, the total scores were categorized in this study as follows: poor (52–90), moderate (91–129), good (130–168), and excellent (169–208) [35]. HPLP-II demonstrated excellent internal consistency, with an overall Cronbach α of 0.94 and subscale reliabilities ranging from 0.68 to 0.89 [18].

### 2.4. Data Collection

Recruitment was conducted at the conclusion of regular theoretical class sessions, during which all eligible students were invited to take part in the study. They were explicitly informed that participation was entirely voluntary and that they could decline or withdraw their consent at any point without providing justification and without any consequences. To reduce self-selection bias, investigators encouraged all students to participate regardless of their lifestyle habits. Questionnaires were self-administered anonymously, and participants were assured of the confidentiality of their responses, which helped minimize social desirability bias. Throughout data collection, investigators were available to assist participants and address any questions or concerns.

### 2.5. Ethical Considerations

The study protocol was reviewed and approved by the University of the Basque Country Ethics Committee for Research Involving Human Subjects (Approval Code: M10/2023/221). Participants received detailed information about the study’s objectives and procedures and the confidentiality of their data. They were also informed that completion of the questionnaire implied their consent to participate.

### 2.6. Data Analysis

Descriptive statistics were used to summarize demographic characteristics and health-promoting lifestyle behaviors. Categorical variables were presented as frequencies and percentages, while continuous variables were described using means and standard deviations. The normality of continuous variables was assessed using the Shapiro–Wilk test in combination with graphical methods (histogram analysis and Q–Q plots). Mean scores were calculated for the overall HPLP-II scale as well as for each of its subscales (adjusting the mean scores for the number of items). For variables that did not follow a normal distribution, non-parametric statistical tests were applied (Mann–Whitney U test and Kruskal–Wallis H test) to ensure analytical robustness. Spearman’s rank correlation coefficients were used to analyze the intercorrelations between subscales to examine relationships between the various dimensions of health-promoting lifestyle behaviors.

Hierarchical multivariate logistic regression analyses were conducted to examine associations between covariates and HPLP-II scores dichotomized as “good” versus “below average.” The original four-category HPLP-II classification (poor, moderate, good, excellent) included very few participants in the “poor” (0.0%) and “excellent” (4.2%) categories, which could compromise statistical reliability. To address this, “good” and “excellent” were combined into a single “good” category and “poor” and “moderate” into a “below average” category. This approach has also been used in a previous study, allowing for meaningful interpretation and comparison with the existing literature [36]. Logistic regression was selected given the categorical nature of the outcome variable. An alternative approach using the HPLP-II score as a continuous variable (e.g., linear regression) was also considered; however, this was not applied in the present study in order to ensure adequate group sizes and stable estimates across categories.

Variables for the hierarchical logistic regression models were selected a priori based on theoretical relevance, the prior literature, and questionnaire availability. Model I included sociodemographic variables, while health-related factors were added to Model II to evaluate their incremental contribution. This approach accounted for potential confounders, assessed model fit, and ensured robust, interpretable results.

After dichotomization, 319 participants (67.0%) were classified as “good” and 157 (33.0%) as “below average.” This distribution ensured that the number of events per variable (EPV) exceeded the recommended threshold of ≥10, supporting stable and reliable estimates. Additionally, the total sample size (*n* = 476) provided adequate statistical power for the hierarchical logistic regression models and helped minimize the risk of overfitting [37].

Model performance was evaluated using chi-square tests, goodness-of-fit indices such as the −2 log-likelihood (−2LL) and Nagelkerke’s R^2^, and the Hosmer–Lemeshow goodness-of-fit test. A likelihood ratio test was used to determine whether Model II significantly improved upon Model I. Multicollinearity among covariates was assessed using variance inflation factors (VIFs), which ranged from 1.02 to 2.80.

Missing data were minimal across variables (1–2 missing values for BMI and age, and 5–6 missing values for maternal and paternal education, respectively; approximately 0.2% to 1.2%). For all analyses, an available-case approach was used, including only participants with complete data on the variables required for each specific analysis. Given the very low proportion of missing data, no imputation procedures were applied, as the impact on the validity of the results or the risk of bias was considered negligible. All analyses were conducted using SPSS version 28.0 (SPSS Inc., Chicago, IL, USA). Statistical significance was set at *p* < 0.05.

## 3. Results

### 3.1. Sociodemographic, Academic, and Health-Related Characteristics of the Participants

A total of 476 students participated in the study, representing a 72.1% response rate among the 660 eligible students. Response rates by academic year were 78.1% for first-year (*n* = 142), 90.6% for second-year (*n* = 145), 76.1% for third-year (*n* = 127), and 37.0% for fourth-year students (*n* = 62).

Among the participants, 81.1% were women, and most were aged 19–20 years (40.1%) or 21–22 years (38.8%). Regarding parental education, a higher proportion of mothers held a university degree (52.8%), whereas fathers more frequently had primary (18.0%) or secondary (45.9%) education only. Fathers also showed a slightly higher employment rate than mothers (85.1% vs. 80.5%). Overall, participants were predominantly characterized by biparental family structures (89.3%), urban residence (92.6%), and living in family-owned housing (92.0%), with 24.4% also employed.

Most students reported no prior experience in the health field (76.9%) and limited participation in university wellness activities (88.9%). Awareness of healthy lifestyles was nearly universal (99.8%), but this was not consistently reflected in practice. Although the majority of students adhered to some healthy behaviors, a small proportion demonstrated risk behaviors, such as smoking (8.0%), low adherence to the Mediterranean diet (4.2%), low levels of physical activity (9.5%), or sleeping fewer than 7 h per night (16.6%). Additionally, 9.9% were overweight, 1.5% were obese, and 13.2% reported having a chronic disease. Most participants exceeded recommended screen time limits on both weekdays (79.2%) and weekends (88.4%) (Table 1).

These findings suggest that although participants generally maintain a healthy lifestyle, there is room for improvement, particularly in reducing sedentary behavior and increasing engagement in health-promoting lifestyle activities.

### 3.2. HPLP-II Scores

The overall HPLP-II score indicated a moderate-to-good level of health-promoting lifestyle behaviors (adjusted mean 2.62 ± 0.33). Among the subscales, interpersonal relations (adjusted mean 3.12 ± 0.44) and spiritual growth (adjusted mean 2.87 ± 0.47) scored highest, whereas health responsibility (adjusted mean 2.20 ± 0.48) and stress management (adjusted mean 2.33 ± 0.44) scored lowest, highlighting these as key areas for improvement. Physical activity (adjusted mean 2.47 ± 0.62) and nutrition (adjusted mean 2.72 ± 0.48) showed intermediate scores (Table 2).

When categorized, the majority of participants demonstrated good levels of health-promoting lifestyle behaviors (62.8%, score: 130–168), one-third exhibited moderate levels (33.0%, score: 91–129), and a small proportion achieved excellent levels (4.2%, score: 169–208). No participants fell into the poor category (0.0%, score: 52–90). The internal consistency of the HPLP-II subscales was good to excellent, with Cronbach’s α ranging from 0.667 to 0.803, and 0.896 for the overall instrument, confirming reliable measurement across all dimensions.

In the bivariate analysis (see Appendix A for detailed comparisons), higher HPLP-II scores were observed among students in the 4th academic year (*p* = 0.016), those with employed parents (mother: *p* = 0.004; father: *p* = 0.034), and students who combined studies with employment (*p* = 0.013). Health-related factors showed stronger associations: sufficient sleep (*p* = 0.024), healthy body weight (*p* = 0.019), higher adherence to the Mediterranean diet (*p* < 0.001), and greater physical activity (*p* < 0.001) were all linked to higher HPLP-II scores, whereas excessive screen time on weekdays was associated with lower HPLP-II scores (*p* = 0.014).

### 3.3. Intercorrelations Between the HPLP-II Subscales

All HPLP-II subscales were significantly correlated, supporting the multidimensional but interconnected nature of health-promoting lifestyle behaviors (Spearman’s r = 0.165–0.564; all *p* < 0.001) (Table 3). Spiritual growth emerged as a central dimension, showing the strongest relationships with overall lifestyle (r = 0.739) and with several other subscales, particularly interpersonal relations (r = 0.564), stress management (r = 0.528), and health responsibility (r = 0.402). In contrast, physical activity showed weaker associations with interpersonal relations (r = 0.165), suggesting it may operate more independently from social dimensions of health behavior. These findings highlight that while improvements in one lifestyle domain may positively influence others, some dimensions—such as physical activity, which showed weaker correlations with social aspects, and health responsibility, which had lower average scores—may differ in how they relate to overall health-promoting lifestyle behaviors.

### 3.4. Factors Influencing the HPLP-II Scores

Table 4 presents the results of the multivariate hierarchical logistic regression analyses of the association between a good HPLP-II score and the sociodemographic–academic and health-related variables.

The inclusion of health-related variables significantly improved the explanatory capacity of the model compared with sociodemographic factors alone, highlighting their greater relevance in predicting health-promoting lifestyle behaviors. Specifically, the −2 log-likelihood decreased from 570.25 in Model I to 496.69 in Model II, and the model chi-square for Model II indicated a significant improvement over Model I (χ^2^ = 93.86, *p* < 0.001). Model II explained 25.4% of the variance in HPLP-II scores (Nagelkerke R^2^ = 0.254), indicating that health-related variables substantially increased the model’s explanatory power. Goodness-of-fit was confirmed using the Hosmer–Lemeshow test, showing adequate fit for Model I (χ^2^ = 6.585, *p* = 0.582) and excellent fit for Model II (χ^2^ = 2.323, *p* = 0.969).

Among the health-related characteristics, adherence to the Mediterranean diet and physical activity emerged as the strongest correlates of a good HPLP-II score after adjusting for confounders. Participants with lower adherence to the Mediterranean diet (medium adherence: AOR = 0.30; 95% CI = 0.19–0.49; *p* < 0.001; low adherence: AOR = 0.06; 95% CI = 0.02–0.20; *p* < 0.001) and lower levels of physical activity (moderate: AOR = 0.52; 95% CI = 0.32–0.85; *p* = 0.008; low: AOR = 0.37; 95% CI = 0.17–0.80; *p* = 0.012) were significantly less likely to exhibit a favorable lifestyle profile compared with participants with higher adherence levels, highlighting these behaviors as key targets for intervention.

Additionally, regarding sociodemographic–academic variables, students who combined studying with employment were more likely to report higher scores on health-promoting lifestyle behaviors (students only studying: AOR = 0.56; 95% CI = 0.32–0.98; *p* = 0.042), suggesting a possible link between structured daily routines and healthier behaviors.

Although the Nagelkerke R^2^ value indicates a moderate explanatory capacity, this is consistent with research on health-promoting lifestyle behaviors, which are influenced by multiple factors not fully captured in the model. Importantly, the model still identifies key correlates, namely Mediterranean diet adherence and physical activity, that provide meaningful insights for interventions aimed at promoting healthy lifestyles.

## 4. Discussion

This study provided a comprehensive assessment of health-promoting lifestyle behaviors among nursing students in Spain using the HPLP-II, while also examining a broad range of associated sociodemographic, academic, and health-related factors. The findings indicated a moderately high or good level of engagement in health-promoting lifestyle behaviors among the participants.

The overall adjusted mean HPLP-II score was 2.62, indicating a relatively high value compared with international reports [8,10,27,36,38,39,40], although some countries reported higher scores [11,22,41]. Considerable variability existed across regions, indicating that strategies to enhance healthy behaviors among nursing students should be tailored to specific national and institutional contexts rather than assuming a universal approach. Scores were notably concentrated in the moderate HPLP-II range (91–129/1.74–2.48), highlighting the need to strengthen education and support to promote healthy lifestyles.

In the present study, the highest adjusted mean subscale scores were observed in interpersonal relations and spiritual growth, consistent with previous studies [8,10,22,36,38,39,40,41]. Interpersonal relations consistently ranked high among nursing students internationally, reflecting strong social connections. Factors such as social norms, peer support, and role models may influence health behaviors and be essential for effective health education [23,42], as well as for understanding patients and managing emotions [43]. These findings suggested that nursing students report strong interpersonal relationships and inner resources that may contribute to their well-being, as suggested by a previous study [26], though causal relationships cannot be established in the present study.

Similarly, spiritual growth might serve as a key coping mechanism and source of internal motivation, relevant both during the academic education process [44,45] and in clinical settings where nurses provide spiritual care [46]. Notably, spiritual growth showed the strongest positive correlation with both interpersonal relations and overall health-promoting lifestyle behaviors, highlighting their potentially close connection in influencing health behaviors and suggesting that they could be addressed together.

Nutrition scored relatively highly (adjusted mean 2.72), suggesting generally favorable dietary habits among Spanish nursing students. This finding is consistent with previous studies reporting moderate-to-high scores in this domain, although variability across contexts has been observed [9,10,11,22,25,27,39,40], and may be partly explained by the influence of the Mediterranean diet, which is culturally embedded in Spain and supported by public health initiatives [47].

In contrast, the lowest scores were observed in physical activity (2.47), stress management (2.33), and health responsibility (2.20), with 9.5% reporting low physical activity. These results align with previous international evidence [9,10,11,22,25,27,39,40] and indicate that these domains remain suboptimal in nursing students. Despite well-known health benefits [48,49], these behaviors appear to be insufficiently prioritized during training.

Linking our findings to the HPM, high scores in interpersonal relations and spiritual growth may suggest that social support and internal motivation facilitate health-enhancing behaviors. In contrast, lower scores in physical activity, stress management, and health responsibility may be influenced by academic, clinical, and environmental factors. These patterns illustrate the interplay between personal, interpersonal, and environmental influences emphasized by the HPM, suggesting that interventions should target both individual behaviors and contextual factors to effectively promote a healthy lifestyle among nursing students.

The hierarchical logistic regression analysis of the factors associated with the HPLP-II score revealed that students’ health-related characteristics significantly predicted their health-promoting lifestyle behaviors when controlling for sociodemographic and academic variables. The inclusion of these health-related characteristics underscores the importance of strengthening such factors among nursing students to encourage healthy behaviors. However, these findings should be interpreted with caution, as some variables—particularly physical activity and nutrition—were included both as components of the HPLP-II (i.e., part of the outcome) and as independent variables in the model. This conceptual overlap may have inflated the observed associations, potentially leading to an overestimation of their strength. Furthermore, the best-fitting model, which incorporated both sociodemographic–academic and health-related variables, explained only 24.5% of the variance in health-promoting lifestyle behaviors, indicating that a substantial proportion of variability remains unexplained. Therefore, in light of these methodological limitations, the findings must be considered carefully.

This study did not find significant associations between health-promoting lifestyle behaviors and several sociodemographic, academic, and health factors, including BMI, age, sex, year of study, prior experience in the health field, participation in university wellness activities, presence of chronic disease, smoking habits, sleep patterns, screen-viewing behaviors, family-related variables, and residence. In contrast, physical activity and adherence to the Mediterranean diet emerged as key correlates associated with healthier lifestyles in this sample. In this regard, physical activity has been identified as a strong correlate in other similar studies of nursing students [27,36], while, to the best of our knowledge, no previous study has examined adherence to the Mediterranean diet as a correlate of health-promoting lifestyle behaviors in this population. It is important to note that the factors that were not significantly associated may not play a major role in this population, or their influence may be context-dependent. Given the cross-sectional design, these associations should be interpreted with caution, and future research is warranted to further explore potential contributing factors.

Importantly, these findings may highlight the role of physical activity and adherence to the Mediterranean diet in promoting healthy lifestyles among nursing students. Although students were well educated on the benefits of physical activity for health promotion, disease prevention, and disease management, a gap remained in their personal self-care practices, particularly regarding exercise. This pattern has been consistently observed internationally [8,39] and has been linked in previous studies to personal and environmental factors such as demanding academic schedules, time constraints, and limited institutional support [24,26,38]. However, these factors were not directly assessed in this study. Nursing students might benefit from support in identifying the underlying reasons for their low physical activity [26]. These findings suggested the need to move beyond purely theoretical instruction and instead emphasize action-oriented learning, as well as the creation of supportive environments that foster healthy nutritional habits and regular physical activity throughout students’ academic journey.

Regarding health-related variables, and consistent with previous studies, this study did not find significant differences in HPLP-II scores among BMI groups (underweight, normal, overweight, and obese) [26,27,36]. Similarly, Kara and İşcan reported that the presence of health problems and smoking habits were not associated with health-promoting lifestyle behaviors [23]. It is possible that the students in this study did not perceive disease as a threat, leaving their health-promoting lifestyle behaviors largely unaffected.

In terms of sociodemographic variables, employment status was a significant correlate of health-promoting lifestyle behaviors in nursing students. Working students might be more likely to adopt health-promoting lifestyle behaviors, possibly due to increased career maturity [50], personal responsibility, and self-awareness gained through professional and academic experiences. These factors collectively support healthier lifestyle choices [51] by increasing students’ health awareness and ability to manage it effectively [52]. Financial independence might also facilitate access to health-related activities and reflect a greater prioritization of well-being, either to sustain job performance or as part of a broader process of self-actualization [40]. However, contrary to our result, no relationship emerged for employment status in a comparable study [27].

The current study did not find any sex difference in the overall HPLP-II score among the nursing students, in concordance with other reports [26,27]. However, other studies have shown disparities associated with this variable [8,36].

Consistent with previous studies, this study did not find a significant association of the HPLP-II score with age [26,27,36] or year of study [8,27]. However, our descriptive analysis showed that the 4th-year students had higher total HPLP-II scores than the other students. Spending more years in nursing education has been associated with improved health-related behaviors [53], as students might develop a stronger sense of health responsibility through exposure to health-promoting content [8,24]. However, such knowledge and responsibility might still be insufficient to fully support consistent engagement in health-promoting lifestyle behaviors [51,54] and self-care.

Finally, this study found no significant association between the family-related variables examined—such as maternal and paternal educational levels, living in the parental home, and family structure—and health-promoting lifestyle behaviors. In line with previous studies, no significant relationship was also identified between the health-promoting lifestyle behaviors and place of residence [23,36] or habitual residence [27].

Limited development of health-promoting lifestyle behaviors in the family setting, under-representation in school curricula, predominantly disease-oriented university programs [25], and barriers such as fatigue, time constraints, and lack of institutional support might hinder participation in health-promoting lifestyle behaviors [55].

However, if nursing students were able to identify the factors that hinder their own health-promoting lifestyle behaviors and learn to manage them, they might be better equipped to encourage and support patients in adopting similar behaviors in the future [54].

### 4.1. Implications for Nursing Education and Practice

Although nursing curricula across Europe and globally include theoretical content on health promotion, translating this knowledge into personal practice remains a challenge for students. Based on the present findings, more structured and behavior-oriented strategies may be needed to bridge this gap, particularly in areas where lower scores were observed, such as physical activity, stress management, and health responsibility.

Given that nursing students are future health professionals and key agents in patient education and health promotion, strengthening their own health behaviors may enhance their effectiveness as role models in clinical practice and improve their ability to promote healthy lifestyles among patients and the wider community. In this regard, universities could consider integrating scheduled physical activity sessions, alongside targeted interventions for stress management within nursing curricula. In addition, behavior-change strategies may help students translate knowledge into sustained habits. Peer-led interventions and mentoring programs may be particularly useful, leveraging interpersonal relationships within nursing cohorts to promote healthy lifestyles. At the institutional level, supportive environments are essential, including access to sports facilities, promotion of Mediterranean diet-aligned food options, and university-wide well-being policies. Tailoring interventions to different academic years may also be beneficial due to varying academic and clinical demands. However, given the cross-sectional design of this study, these implications should be interpreted with caution, as causal relationships cannot be inferred.

Given the paucity of research on factors influencing healthy behaviors among nursing students in Spain, this study contributes to the development of culturally sensitive strategies to promote such behaviors. Establishing supportive environments that encourage healthy lifestyles may better prepare students not only for their own well-being but also for their future professional role as health promoters in clinical settings. Our findings suggest that adherence to a Mediterranean diet and higher levels of physical activity are important correlates of healthier lifestyles in this sample, although the model accounted for only a moderate proportion of variance.

Future research should explore additional environmental, behavioral, psychological, academic, and institutional factors, such as family and cultural norms, time management, health literacy, stress and coping mechanisms, self-efficacy, motivation, exposure to nursing role models, clinical experiences, institutional support, and access to health-related resources. Conducting qualitative studies to identify barriers to and facilitators of health-promoting lifestyle behaviors would further enhance predictive models and provide a more comprehensive understanding of the determinants of nursing students’ health behaviors, supporting the design of effective interventions.

### 4.2. Study Limitations

This study has several limitations. Its cross-sectional design prevented causal inference and the assessment of changes in health-promoting lifestyle behaviors over time. All variables were self-reported, which might have introduced recall and social desirability biases; in particular, nursing students might have over-reported healthy behaviors, potentially inflating observed associations. Data collection at a single institution using convenience sampling might have limited generalizability, and voluntary participation could have led to selection bias, with students more sensitive to health issues being over-represented. Lower participation among fourth-year students might have further limited the applicability of findings to students in their final year, which might be related to reduced attendance in theoretical classes. HPLP-II scores were dichotomized from their original four-category classification, which might have resulted in some loss of information, although this allowed for more robust analyses and comparisons with previous studies. Alternative modeling strategies could be considered in future research to capture the full variability of health-promoting lifestyle behaviors. Some potentially important variables (e.g., mental health or socioeconomic status) were not included and may contribute to unexplained variability in health-promoting lifestyle behaviors. Finally, construct overlap existed because domains such as physical activity and nutrition were used both as components of the HPLP-II outcome and as associated variables, which could inflate associations and limit interpretability. Future research could address this by using alternative outcome measures, separating associated variables from outcome domains, or employing advanced statistical methods. Longitudinal and multicenter designs should also be adopted in future studies to gain a deeper understanding of the evolution and broader patterns of health-promoting lifestyle behaviors among nursing students.

Despite these limitations, this study provided a valuable, in-depth assessment of the HPLP-II score and its associations with a wide range of sociodemographic, academic, and health-related variables. With a robust hierarchical logistic regression model, the findings contributed meaningfully to the existing literature and highlighted critical areas for targeted intervention. Furthermore, this study demonstrated the excellent reliability of the HPLP-II scores within the population of nursing students in Spain.

## 5. Conclusions

In this study, the majority of the nursing students exhibited moderate-to-good levels of health-promoting lifestyle behaviors. Among the subdomains, interpersonal relations and spiritual growth received the highest scores, while health responsibility and stress management scored the lowest. Notably, spiritual growth was strongly correlated with interpersonal relations, stress management, and health responsibility. Health responsibility was also strongly correlated with stress management at the individual level, suggesting a personal influence on health-promoting lifestyle behaviors.

The health-related variables showed stronger associations with health-promoting lifestyle behaviors than the sociodemographic–academic variables. In particular, adherence to the Mediterranean diet and engagement in physical activity as health-related factors—along with employment status as a sociodemographic variable—were significantly associated with health-promoting lifestyle behaviors.

These results suggest that enhancing the nursing curriculum and training environment may support nutrition, physical activity, and other health-promoting lifestyle behaviors. Such efforts could help to nursing students manage their own health, act as role models for healthy lifestyles, and educate the public in their future professional roles.

## Figures and Tables

**Table 1:** Sociodemographic–academic and health-related variables of the participants (*n* = 476).

			*n* (%)
Sex		Female	386 (81.1)
		Male	90 (18.9)
		Non-binary/NA	0 (0)
Age, year Missing values: 2		17–18	22 (4.6)
		19–20	190 (40.1)
		21–22	184 (38.8)
		>22	78 (16.5)
		Min–max	17.79–27.81
		Mean ± SD	20.48 ± 1.87
Year of study		1st year	142 (29.8)
		2nd year	145 (30.5)
		3rd year	127 (26.7)
		4th year	62 (13.0)
Maternal educational level Missing values: 6		Primary education	66 (14.0)
		Secondary education	156 (33.2)
		Higher education	248 (52.8)
Paternal educational level Missing values: 5		Primary education	85 (18.0)
		Secondary education	216 (45.9)
		Higher education	170 (36.1)
Mother’s employment status		Yes	383 (80.5)
		No	93 (19.5)
Father’s employment status		Yes	405 (85.1)
		No	71 (14.9)
Family structure		Monoparental	51 (10.7)
		Biparental	425 (89.3)
Place of residence		Urban	441 (92.6)
		Rural	35 (7.4)
Habitual residence		Family home	438 (92.0)
		Residence or flat renting	38 (8.0)
Employment status		Yes	116 (24.4)
		No	360 (75.6)
Previous experience in the health field		Yes	110 (23.1)
		No	366 (76.9)
Participation in university wellness activities		Yes	53 (11.1)
		No	423 (88.9)
Awareness about health-promoting lifestyle		Yes	475 (99.8)
		No	1 (0.2)
Chronic disease		Yes	63 (13.2)
		No	413 (86.8)
Smoking status		Yes	38 (8.0)
		No	438 (92)
Sleep duration		≥7 h/day	397 (83.4)
		<7 h/day	79 (16.6)
		Min–max	5–10
		Mean ± SD	7.2 ± 0.79
BMI Missing values: 1		Underweight	43 (9.1)
		Healthy weight	378 (79.6)
		Overweight	47 (9.9)
		Obesity	7 (1.5)
		Min–max	16.13–33.14
		Mean ± SD	21.64 ± 2.84
KIDMED result (Mediterranean diet adherence)		Low adherence	20 (4.2)
		Medium adherence	231 (48.5)
		High adherence	225 (47.3)
		Min–max	1–12
		Mean ± SD	7.22 ± 2.07
IPAQ result (physical activity level)		Low level	45 (9.5)
		Moderate level	164 (34.5)
		High level	267 (56.1)
Total screen-viewing duration	Weekday	≤3 h/day	100 (21.0)
		>3 h/day	376 (79.2)
		Min–max	0.37–19.00
		Mean ± SD	5.52 ± 2.96
	Weekend day	≤3 h/day	55 (11.6)
		>3 h/day	421 (88.4)
		Min–max	0.33–21.00
		Mean ± SD	6.76 ± 3.17

Abbreviations: NA = no answer; SD = standard deviation.

**Table 2:** Health-Promoting Lifestyle Profile II scores of the participants (*n* = 476).

Dimension/Subscale	Mean Score ± SD	Adjusted Mean Score (Mean/Number of Items) ± SD	Minimum–Maximum Score Recorded	Cronbach’s α	Item
Health responsibility	19.83 ± 4.39	2.20 ± 0.48	10–34/1.11–3.77	0.763	9
Physical activity	19.76 ± 4.95	2.47 ± 0.62	8–32/1.0–4.0	0.758	8
Nutrition	24.50 ± 4.35	2.72 ± 0.48	12–36/1.33–4.0	0.667	9
Spiritual growth	25.85 ± 4.25	2.87 ± 0.47	9–36/1.0–4.0	0.803	9
Interpersonal relations	28.09 ± 3.97	3.12 ± 0.44	14–36/1.55–4.0	0.776	9
Stress management	19.65 ± 3.52	2.33 ± 0.44	8–29/1.00–3.62	0.669	8
Overall lifestyle	136.67 ± 3.52	2.62 ± 0.33	92–194/1.76–3.73	0.896	52

Abbreviation: SD = standard deviation.

**Table 3:** Intercorrelations between HPLP-II subscales among the participants (*n* = 476).

	Health Responsibility	Physical Activity	Nutrition	Spiritual Growth	Interpersonal Relations	Stress Management	Overall Lifestyle
Health Responsibility	1	0.272 ***	0.359 ***	0.402 ***	0.357 ***	0.360 ***	0.654 ***
Physical Activity		1	0.488 ***	0.317 ***	0.165 ***	0.352 ***	0.665 ***
Nutrition			1	0.258 ***	0.215 ***	0.292 ***	0.655 ***
Spiritual Growth				1	0.564 ***	0.528 ***	0.739 ***
Interpersonal Relations					1	0.306 ***	0.605 ***
Stress Management						1	0.655 ***
Overall Lifestyle							1

*** *p* < 0.001; Spearman’s rank correlation coefficients.

**Table 4:** Hierarchical logistic regression analysis of the sociodemographic–academic and health-related characteristics predicting the HPLP-II score among the participants (*n* = 476).

Variable		Model I		Model II	
		AOR (95% CI)	*p* Value	AOR (95% CI)	*p* Value
Sociodemographic–academic characteristics					
Sex	Female	Ref	-	Ref	-
	Male	0.64 (0.37–1.09)	0.097	0.68 (0.37–1.24)	0.214
Age, year	>22	Ref	-	Ref	-
	21–22	0.82 (0.44–1.51)	0.524	0.48 (0.21–1.09)	0.081
	19–20	0.79 (0.38–1.62)	0.514	0.44 (0.16–1.25)	0.124
	17–18	0.75 (0.23–2.40)	0.629	0.50 (0.11–2.27)	0.371
Year of study	4th year	Ref	-	Ref	-
	3rd year	0.52 (0.25–1.08)	0.078	0.58 (0.25–1.30)	0.189
	2nd year	0.58 (0.26–1.27)	0.174	0.68 (0.27–1.70)	0.411
	1st year	0.53 (0.23–1.23)	0.139	0.70 (0.26–1.88)	0.477
Maternal educational level	Higher education	Ref	-	Ref	-
	Secondary education	0.63 (0.40–1.01)	0.056	0.67 (0.40–1.26)	0.133
	Primary education	0.59 (0.30–1.14)	0.118	0.79 (0.37–1.66)	0.531
Paternal educational level	Higher education	Ref	-	Ref	-
	Secondary education	0.96 (0.60–1.54)	0.871	1.06 (0.62–1.80)	0.839
	Primary education	1.26 (0.67–2.35)	0.477	1.58 (0.77–3.24)	0.211
Mother’s employment status	Yes	Ref	-	Ref	-
	No	0.70 (0.43–1.17)	0.174	0.71 (0.40–1.27)	0.234
Father’s employment status	Yes	Ref	-	Ref	-
	No	0.79 (0.43–1.44)	0.433	0.80 (0.40–1.56)	0.514
Family structure	Monoparental	Ref	-	Ref	-
	Biparental	1.09 (0.55–2.17)	0.798	0.93 (0.42–2.04)	0.858
Place of residence	Urban	Ref	-	Ref	-
	Rural	0.89 (0.40–1.94)	0.762	0.91 (0.38–2.17)	0.834
Habitual residence	Family home	Ref	-	Ref	-
	Residence or flat renting	1.07 (0.51–2.25)	0.854	1.14 (0.49–2.65)	0.750
Employment status	Yes	Ref	-	Ref	-
	No	0.64 (0.39–1.06)	0.081	0.56 (0.32–0.98)	0.042
Health-related characteristics					
Previous experience in the health field	Yes			Ref	-
	No			1.70 (0.83–3.47)	0.149
Participation in university wellness activities	Yes			Ref	-
	No			0.83 (0.39–1.77)	0.634
Chronic disease	Yes			Ref	-
	No			0.93 (0.48–1.80)	0.835
Smoking status	Yes			Ref	-
	No			1.63 (0.73–3.62)	0.232
Sleep duration	≥7 h/day			Ref	-
	<7 h/day			0.64 (0.35–1.18)	0.157
BMI	Underweight			Ref	-
	Healthy weight			1.18 (0.54–2.61)	0.678
	Overweight			1.11 (0.39–3.15)	0.844
	Obesity			0.13 (0.01–1.52)	0.105
KIDMED result (Mediterranean diet adherence)	High adherence			Ref	-
	Medium adherence			0.30 (0.19–0.49)	<0.001
	Low adherence			0.06 (0.02–0.20)	<0.001
IPAQ result (Physical activity level)	High level			Ref	-
	Moderate level			0.52 (0.32–0.85)	0.008
	Low level			0.37 (0.17–0.80)	0.012
Screen-viewing duration (weekday)	≤3 h/day			Ref	-
	>3 h/day			1.49 (0.80–2.78)	0.209
Screen-viewing duration (weekend day)	≤3 h/day			Ref	-
	>3 h/day			0.87 (0.38–1.96)	0.734
Model chi-square		20.31 (*p* = 0.258)		93.86 (*p* < 0.001)	
−2 log-likelihood		570.25		496.69	
Degree of freedom		17		31	
Nagelkerke R^2^		0.059		0.254	
Likelihood ratio test				73.55 (*p* < 0.001)	
Hosmer–Lemeshow test		6.585 (*p* = 0.582)		2.323 (*p* = 0.969)	

Abbreviations: AOR = Adjusted Odds Ratio; CI = Confidence Interval.

## Data Availability

The original data presented in the study are openly available in the Harvard Dataverse repository under the title “Replication Data for: Nursing Students HPLP_II Spain” at https://doi.org/10.7910/DVN/UPXF3K (accessed on 12 February 2026). All data were rigorously anonymized prior to their public release.

## Public Involvement Statement

No public involvement in any aspect of this research.

## Guidelines and Standards Statement

This manuscript was prepared in accordance with the STROBE (Strengthening the Reporting of Observational Studies in Epidemiology) Statement for reporting cross-sectional studies [56].

## Abbreviations

The following abbreviations are used in this manuscript:

−2LL	−2 log-likelihood
AOR	Adjusted Odds Ratio
BMI	Body Mass Index
CGE	Consejo General de Enfermería/General Council of Nursing
CI	Confidence Interval
HPLP-II	Health-Promoting Lifestyle Profile-II
HPM	Health Promotion Model
IPAQ	International Physical Activity Questionnaire
KIDMED	Adherence to the Mediterranean diet questionnaire
NCD	Non-communicable diseases
STROBE	Strengthening the Reporting of Observational Studies in Epidemiology
USA	United States of America
WHO	World Health Organization

## Appendix A

**Table A1:** Comparison of HPLP II among students in terms of sociodemographic–academic and health-related characteristics (n = 476).

Variable (N)		HPLPII Dimension TOTAL	
		Mean (SD); 95% CI	Test Results Z *p*
Sex ^a^	Female (386)	2.61 (0.32); 2.57–2.64	−1.861 0.063
	Male (90)	2.70 (0.37); 2.62–2.78	
Age, year ^b^ Missing values: 2	17–18 (22)	2.49 (0.28); 2.36–2.62	5.673 0.129
	19–20 (188)	2.61 (0.30); 2.56–2.65	
	21–22 (179)	2.66 (0.38); 2.60–2.71	
	22+ (76)	2.63 (0.29); 2.56–2.69	
Year of study ^b^	1st year (142)	2.57 (0.31); 2.52–2.63	10.392 0.016
	2nd year (145)	2.64 (0.31); 2.58–2.69	
	3rd year (127)	2.62 (0.35); 2.56–2.69	
	4th year (62)	2.72 (0.40); 2.62–2.82	
Maternal education level ^b^ Missing values: 6	Primary education (66)	2.57 (0.34); 2.49–2.65	3.241 0.198
	Secondary education (156)	2.62 (0.35); 2.56–2.67	
	Higher education (248)	2.64 (0.32); 2.60–2.69	
Paternal education level ^b^ Missing values: 5	Primary education (85)	2.62 (0.35); 2.54–2.70	2.465 0.292
	Secondary education (216)	2.60 (0.35); 2.56–2.65	
	Higher education (170)	2.65 (0.30); 2.60–2.70	
Mother’s employment status ^a^	Yes (383)	2.65 (0.33); 2.61–2.68	−2.843 0.004
	No (93)	2.54 (0.33); 2.47–2.61	
Father’s employment status ^a^	Yes (405)	2.64 (0.34); 2.60–2.67	−2.120 0.034
	No (71)	2.55 (0.29); 2.48–2.62	
Family structure ^a^	Monoparental (51)	2.57 (0.35); 2.47–2.68	−1.145 0.252
	Biparental (425)	2.63 (0.33); 2.60–2.66	
Place of Residence ^a^	Urban (441)	2.63 (0.34); 2.60–2.66	−0.165 0.869
	Rural (35)	2.56 (0.25); 2.47–2.65	
Habitual Residence ^a^	Family house (438)	2.62 (0.34); 2.59–2.66	−1.087 0.277
	Residence or flat renting (38)	2.62 (0.30); 2.52–2.72	
Employment status ^a^	Yes (116)	2.70 (0.33); 2.64–2.76	−2.479 0.013
	No (360)	2.60 (0.33); 2.56–2.63	
Previous experience in the health field ^a^	Yes (110)	2.58 (0.29); 2.52–2.64	−1.782 0.075
	No (366)	2.64 (0.34); 2.60–2.67	
Participation in university wellness activities ^a^	Yes (53)	2.65 (0.35); 2.55–2.75	−1.066 0.286
	No (423)	2.62 (0.34); 2.59–2.65	
Chronic disease ^a^	Yes (63)	2.64 (0.36); 2.54–2.73	−0.219 0.826
	No (413)	2.62 (0.33); 2.59–2.66	
Smoking status ^a^	Yes (38)	2.57 (0.31); 2.47–2.67	−0.882 0.378
	No (438)	2.63 (0.34); 2.60–2.66	
Sleep duration ^a^	≥7 h/day (397)	2.64 (0.34); 2.61–2.67	−2.255 0.024
	<7 h/day (79)	2.55 (0.32); 2.48–2.62	
BMI ^b^ Missing values: 1	Underweight (43)	2.56 (0.33); 2.45–2.66	9.960 0.019
	Healthy weight (378)	2.63 (0.33); 2.60–2.66	
	Overweight (47)	2.69 (0.37); 2.58–2.79	
	Obesity (7)	2.25 (0.35); 1.92–2.58	
KIDMED ^b^ result (Mediterranean diet adherence)	Low (20)	2.28 (0.23); 2.17–2.39	62.675 <0.001
	Medium (231)	2.54 (0.31); 2.50–2.58	
	High (225)	2.74 (0.33); 2.70–2.78	
IPAQ ^b^ result (Physical activity level)	Low (45)	2.50 (0.38); 2.39–2.62	32.436 <0.001
	Moderate (164)	2.54 (0.31); 2.49–2.58	
	High (267)	2.70 (0.33); 2.66–2.74	
Screen-viewing duration (weekday) ^a^	≤3 h/day (100)	2.70 (0.39); 2.62–2.78	−2.447 0.014
	>3 h/day (376)	2.61 (0.32); 2.57–2.64	
Screen-viewing duration (weekend day) ^a^	≤3 h/day(55)	2.68 (0.35); 2.58–2.78	−1.562 0.118
	>3 h/day (421)	2.62 (0.33); 2.59–2.65	

^a^ Mann–Whitney U test. ^b^ Kruskal–Wallis H test. Abbreviations: SD = standard deviation; CI = Confidence Interval.

## References

[1] World Health Organization (2020). State of the World’s Nursing 2020: Investing in Education, Jobs and Leadership.

[2] White J., Gunn M., Chiarella M., Catton H., Stewart D. (2025). Renewing the Definitions of Nursing and a Nurse.

[3] Global Burden of Disease Collaborative Network Global Burden of Disease Study 2021 Results. https://vizhub.healthdata.org/gbd-results/.

[4] National Statistics Institute Statistics on Deaths by Cause of Death 2024. https://www.ine.es/dyngs/Prensa/pEDCM2024.htm.

[5] World Health Organization (2021). WHO Discussion Paper for the Regional Expert Consultations: Development of an Implementation Roadmap 2023–2030 for the Global Action Plan for the Prevention and Control of NCDs 2013–2030.

[6] Puente-Hidalgo S., Prada-García C., Benítez-Andrades J.A., Fernández-Martínez E. (2024). Promotion of healthy habits in university students: Literature review. Healthcare.

[7] Martin S.D., Urban R.W., Johnson A.H., Magner D., Wilson J.E., Zhang Y. (2022). Health-related behaviors, self-rated health, and predictors of stress and well-being in nursing students. J. Prof. Nurs..

[8] Hwang Y., Oh J. (2020). Factors affecting health-promoting behaviors among nursing students. Int. J. Environ. Res. Public Health.

[9] Polat Ü., Özen Ş., Kahraman B.B., Bostanoğlu H. (2016). Factors affecting health-promoting behaviors in nursing students at a university in Turkey. J. Transcult. Nurs..

[10] Mak Y.W., Kao A.H.F., Tam L.W.Y., Tse V.W.C., Tse D.T.H., Leung D.Y.P. (2018). Health-promoting lifestyle and quality of life among Chinese nursing students. Prim. Health Care Res. Dev..

[11] Fashafsheh I., Al-Ghabeesh S.H., Ayed A., Salama B., Batran A., Bawadi H. (2021). Health-promoting behaviors among nursing students: Palestinian perspective. Inquiry.

[12] Gipson C.S., Deal B., Skinner M. (2024). Examining well-being and healthy lifestyle interventions among nursing students worldwide: A scoping review. J. Holist. Nurs..

[13] Chen C., Meier S.T. (2021). Burnout and depression in nurses: A systematic review and meta-analysis. Int. J. Nurs. Stud..

[14] General Council of Nursing (2024). Study on the Impact of Healthcare Pressure on the Nursing Profession 2024.

[15] Murdaugh C.L., Parsons M.A., Pender N.J. (2021). Health Promotion in Nursing Practice.

[16] Kumar S., Preetha G. (2012). Health promotion: An effective tool for global health. Indian J. Community Med..

[17] Walker S.N., Sechrist K.R., Pender N.J. (1987). The health-promoting lifestyle profile: Development and psychometric characteristics. Nurs. Res..

[18] Zambrano Bermeo R.N., Estrada González C., Herrera Guerra E.d.P., Avilés González C.I. (2024). Reliability and validity of the health-promoting lifestyle profile II Spanish version in university students. Healthcare.

[19] Mohamadian H., Ghannaee M., Kortdzanganeh J., Meihan L. (2013). Reliability and construct validity of the Iranian version of health-promoting lifestyle profile in a female adolescent population. Int. J. Prev. Med..

[20] Savarese G., Carpinelli L., Cavallo P., Vitale M.P. (2018). Italian psychometric validation of the multidimensional students’ health-promoting lifestyle profile scale. Health.

[21] Cao W., Guo Y., Ping W., Zheng J. (2016). Development and psychometric tests of a Chinese version of the HPLP-II scales. Chin. J. Dis. Control.

[22] Davis B., Badr L.K., Doumit R. (2002). Health-promoting behaviors among American and Lebanese nursing students. Worldviews Evid. Based Nurs..

[23] Kara B., İşcan B. (2016). Predictors of Health Behaviors in Turkish Female Nursing Students. Asian Nurs. Res..

[24] Can G., Ozdilli K., Erol O., Unsar S., Tulek Z., Savaser S., Ozcan S., Durna Z. (2008). Comparison of the health-promoting lifestyles of nursing and non-nursing students in Istanbul, Turkey. Nurs. Health Sci..

[25] Dural G., Aktürk Ü. (2021). Factors affecting healthy lifestyle behaviors of nursing students. Anatol. J. Health Res..

[26] Baykal D., Kutlu L., Demir B.D. (2022). The correlation between nursing students’ healthy lifestyle behaviors, cardiovascular disease risk factors’ knowledge level, and obsession symptoms. J. Educ. Health Promot..

[27] Doumit R., Habre M., Cattan R., Abi Kharma J., Davis B. (2022). Health-promoting behaviors and self-efficacy among nursing students in times of uncertainty. Worldviews Evid. Based Nurs..

[28] Serra-Majem L., Ribas L., Ngo J., Ortega R.M., García A., Pérez-Rodrigo C., Aranceta J. (2007). Food, youth and the Mediterranean diet in Spain. Development of KIDMED, Mediterranean diet quality index in children and adolescents. Public Health Nutr..

[29] Craig C.L., Marshall A.L., Sjöström M., Bauman A.E., Booth M.L., Ainsworth B.E., Pratt M., Ekelund U., Yngve A., Sallis J.F. (2003). International physical activity questionnaire: 12-country reliability and validity. Med. Sci. Sports Exerc..

[30] Sapra R.L. (2022). How to calculate an adequate sample size. How to Practice Academic Medicine and Publish from Developing Countries.

[31] Watson N.F., Badr M.S., Belenky G., Bliwise D.L., Buxton O.M., Buysse D., Dinges D.F., Gangwisch J., Grandner M.A., Kushida C. (2015). Recommended amount of sleep for a healthy adult: A joint consensus statement of the American academy of sleep medicine and sleep research society. Sleep.

[32] Ross R., Chaput J.P., Giangregorio L.M., Janssen I., Saunders T.J., Kho M.E., Poitras V.J., Tomasone J.R., El-Kotob R., McLaughlin E.C. (2020). Canadian 24-hour movement guidelines for adults aged 18-64 years and adults aged 65 years or older. Appl. Physiol. Nutr. Metab..

[33] Centers for Disease Control and Prevention Adult BMI Categories. https://www.cdc.gov/bmi/adult-calculator/bmi-categories.html.

[34] International Physical Activity Questionnaire. IPAQ Scoring Protocol. https://sites.google.com/view/ipaq.

[35] Alzahrani S.H., Malik A.A., Bashawri J., Shaheen S.A., Shaheen M.M., Alsaib A.A., Mubarak M.A., Adam Y.S., Abdulwassi H.K. (2019). Health-promoting lifestyle profile and associated factors among medical students in a Saudi university. SAGE Open Med..

[36] Gurusamy J., Amudhan S., Veerabhadraiah K.B., Palaniappan M. (2022). Health-promoting behaviours, their relationships and correlates in nursing students: Implications for nursing education and practice. J. Prof. Nurs..

[37] Peduzzi P., Concato J., Kemper E., Holford T.R., Feinstein A.R. (1996). A simulation study of the number of events per variable in logistic regression analysis. J. Clin. Epidemiol..

[38] Al-Kandari F., Vidal V.L., Thomas D. (2008). Health-promoting lifestyle and body mass index among college of nursing students in Kuwait: A correlational study. Nurs. Health Sci..

[39] Hosseini M., Ashktorab T., Taghdisi M.H., Vardanjani A.E., Rafiei H. (2014). Health-promoting behaviors and their association with certain demographic characteristics of nursing students of Tehran City in 2013. Glob. J. Health Sci..

[40] Hui W.H. (2002). The health-promoting lifestyles of undergraduate nurses in Hong Kong. J. Prof. Nurs..

[41] Hayati Ifroh R., Imamah I.N., Rizal A.A.F. (2022). The Health-Promoting Lifestyle Assessment Among Nursing Students In East Kalimantan. J. Ilmu Kesehat. Masy..

[42] Lane I.F. (2010). Professional competencies in health sciences education: From multiple intelligences to the clinic floor. Adv. Health Sci. Educ. Theory Pract..

[43] Kawashima T., Ota Y., Aikawa G., Watanabe M., Ashida K., Sakuramoto H. (2025). Effectiveness of emotional intelligence training on nurses’ and nursing students’ emotional intelligence, resilience, stress, and communication skills: A systematic review and meta-analysis. Nurse Educ. Today.

[44] Felicilda-Reynaldo R.F.D., Cruz J.P., Papathanasiou I.V., Helen Shaji J.C., Kamau S.M., Adams K.A., Valdez G.F.D. (2019). Quality of life and the predictive roles of religiosity and spiritual coping among nursing students: A multi-country study. J. Relig. Health.

[45] Kurtgöz A., Koç Z. (2025). Nursing students’ spiritual/religious coping strategies dealing with first experience of witnessing death during clinical practices. Omega.

[46] Eskandari N., Golaghaie F., Aghabarary M., Dinmohammadi M., Koohestani H., Didehdar M., Dehghankar L., Abbasi M. (2019). Explaining the relationship between moral intelligence and professional self-concept with the competency of nursing students in providing spiritual care to promote nursing education. J. Educ. Health Promot..

[47] Spanish Agency for Food Safety and Nutrition Nutrition and Physical Activity Campaigns. https://www.aesan.gob.es/.

[48] Kramer A., Xiao J. (2020). An overview of the beneficial effects of exercise on health and performance. Physical Exercise for Human Health.

[49] Nacar M., Baykan Z., Cetinkaya F., Arslantas D., Ozer A., Coskun O., Bati H., Karaoglu N., Elmali F., Yilmaze G. (2014). Health promoting lifestyle behaviour in medical students: A multicentre study from Turkey. Asian Pac. J. Cancer Prev..

[50] Savickas M.L., Porfeli E.J. (2011). Revision of the career maturity inventory: The adaptability form. J. Career Assess..

[51] Zhang Z., Abdullah H., Ghazali A.H.A., D’Silva J.L., Ismail I.A., Huang Z. (2024). The influence of health awareness on university students’ healthy lifestyles: The chain mediating role of self-esteem and social support. PLoS ONE.

[52] Lange T. (2021). Job satisfaction and implications for organizational sustainability: A resource efficiency perspective. Sustainability.

[53] Callaghan P. (1995). A preliminary survey of nurses’ health-related behaviours. Int. J. Nurs. Stud..

[54] Bryer J., Cherkis F., Raman J. (2013). Health-promotion behaviors of undergraduate nursing students: A survey analysis. Nurs. Educ. Perspect..

[55] Ross A., Bevans M., Brooks A.T., Gibbons S., Wallen G.R. (2017). Nurses and health-promoting behaviors: Knowledge may not translate into self-care. AORN J..

[56] Von Elm E., Altman D.G., Egger M., Pocock S.J., Gøtzsche P.C., Vandenbroucke J.P. (2007). The Strengthening the Reporting of Observational Studies in Epidemiology (STROBE) statement: Guidelines for reporting observational studies. Lancet.

